# Potent Phosphodiesterase Inhibition and Nitric Oxide Release Stimulation of Anti-Impotence Thai Medicinal Plants from “MANOSROI III” Database

**DOI:** 10.1155/2017/9806976

**Published:** 2017-07-25

**Authors:** Aranya Manosroi, Theeraphong Tangjai, Charinya Chankhampan, Worapaka Manosroi, Yaravee Najarut, Worapong Kitdamrongtham, Jiradej Manosroi

**Affiliations:** ^1^Manosé Health and Beauty Research Center, Chiang Mai 50200, Thailand; ^2^Faculty of Science and Technology, North-Chiang Mai University, Chiang Mai 50230, Thailand; ^3^Research Administration Center, Chiang Mai University, Chiang Mai 50200, Thailand; ^4^Faculty of Pharmacy, Payap University, Chiang Mai 50000, Thailand; ^5^Faculty of Medicine, Chiang Mai University, Chiang Mai 50200, Thailand

## Abstract

Seven plants in the top rank were selected from the “MANOSROI III” database using the two Thai keywords which meant impotence and sexual tonic.* Boesenbergia rotunda* (L.) Mansf. extract [EDP1-001(1)] gave the highest PDE inhibition activity of 4.36-fold sildenafil, a standard anti-impotence drug.* Plumbago indica* Linn. extract [EDP2-001(1)] exhibited the highest NO release stimulation activity of 666.85% which was 1.50-fold acetylcholine, a standard drug. Most selected plant extracts were nontoxic to EA.hy926 cells at 1.0 mg/mL. EDP1-001(1) exhibited the LD_50_ value of acute oral toxicity in male ICR mice of over 5,000 mg/kg body weight. EDP1-001(1) also indicated the improvement of sexual behaviors in the paroxetine-induced sexual dysfunction male mice with the evaluation of number of courtships (NC), mount frequency (MF), intromission frequency (IF), and ejaculatory frequency (EF) at 87.67 ± 6.17, 121.00 ± 23.50, 36.00 ± 3.21, and 13.67 ± 2.96 which were 2.63-, 1.27-, 0.53-, and 0.62-fold sildenafil-treated mice at day 14 of the treatments, respectively. The present study has not only confirmed the traditional use of Thai plants for the treatment of ED but also indicated the potential and application of the “MANOSROI III” database for Thai plant selection to be developed as ED food supplements.

## 1. Introduction

Erectile dysfunction (ED) or impotence is a sexual disorder of men who fail to accomplish or maintain an adequate erection of penis to allow the pleasing of sexual intercourse. It is regarded as a major health problem of the ageing population and can cause considerable distress, unhappiness, and relationship problems [1]. Phosphodiesterases (PDEs) are classes of enzymes that are capable of cleaving the phosphodiester both in cyclic adenosine monophosphate (cAMP) and in cyclic guanosine monophosphate (cGMP), which are the important intracellular messengers to stimulate the penile smooth muscle relaxation and generate the penile erection. Sildenafil, the first effective oral drug, belongs to the group called selective phosphodiesterase type 5 (PDE-5) inhibitors. This drug preserves the cAMP and cGMP levels by inhibiting the action of phosphodiesterase enzymes, thereby enhancing and prolonging the penile erection. However, it may cause moderate to serious side effects, such as headache, facial flushing, dyspepsia, visual disturbance, heart attack, or stroke and even death [2–4]. Nitric oxide (NO) released from the autonomic nerve terminals supplying the penis and the endothelium of penile vascular structures is a primary neurotransmitter that plays a crucial role in penile erection. It activates the soluble guanylyl cyclase enzyme to produce cGMP, resulting in the stimulation of penile smooth muscle relaxation and erection of the penis. NO signaling pathway is now understood to be a novel and promising therapy for ED [4–6].

The Thai medicinal plant recipe database “MANOSROI III” has been developed since 1993 by Professor Dr. Jiradej Manosroi. The recipes have been collected, interpreted, and recorded in this database. This database contains the Thai medicinal plant recipes for pharmaceuticals, cosmetics, and food supplements from all regions of Thailand of over 89,000 recipes with more than 8,300 medicinal plants, as the folklore wisdom treatments of several diseases and symptoms including ED. Many research outcomes have been produced from this database. Several Thai medicinal plant recipes as well as Thai medicinal plants contained in the recipes have long been traditionally used for enhancing sexual performance. In this present study, the two Thai keywords including “Sueam Sa Mat Tha Phap Thang Phet” and “Bam Rung Kam Lang Thang Phet” which meant impotence and sexual energizer, respectively, have been used to search for the target ED plants from this database. The PDE inhibition and NO release stimulation as well as the phytochemicals and the in vivo acute toxicity of the extracts from the selected plants were investigated. In addition, sexual behavior stimulatory effects of the selected extract were evaluated on paroxetine-induced sexual dysfunction male mice. The results from this study can confirm the traditional use for ED which will be beneficial for the further development of the selected plant as a novel drug for ED treatment.

## 2. Materials and Methods

### 2.1. Materials and Chemicals

The PDE activity assay kit containing the PDE enzyme from bovine brain, 5′-nucleotidase, 3′,5′-cGMP substrate, PDE assay buffer, and green assay reagent were purchased from Abcam (Cambridge, MA, USA). Sildenafil citrate, acetylcholine chloride, N_*ω*_-nitro-L-arginine methyl ester hydrochloride (L-NAME), sulforhodamine B (SRB), Griess reagent, 17*α*-ethinylestradiol, and progesterone were obtained from Sigma-Aldrich (St. Louis, MO, USA). Dulbecco's Modified Eagle Medium (DMEM) and fetal bovine serum (FBS) were from Gibco®, Life Technologies Corporation (NY, USA). DMEM-no phenol red was from HyClone™, GE Healthcare Life Sciences (UT, USA). Paroxetine hydrochloride was obtained from GlaxoSmithKline (Bangkok, Thailand). All other chemicals and reagents were of analytical grade.

### 2.2. Selection of Plants from the Thai Medicinal Plant Recipe Database “MANOSROI III”

The aphrodisiac medicinal plants were searched from the Thai medicinal plant recipe database “MANOSROI III” using the two Thai keywords of “Sueam Sa Mat Tha Phap Thang Phet” and “Bam Rung Kam Lang Thang Phet” which meant impotence and sexual tonic, respectively. The searched plants were coded with EDP1 and EDP2 for “Sueam Sa Mat Tha Phap Thang Phet” and “Bam Rung Kam Lang Thang Phet,” respectively. To select the plants, the plants were ranked by frequencies of appearing in the recipes and coded with 001, 002, 003, and so forth, respectively. The plants which were in the top three from each of the searched keywords with the total of 6 plants were selected for this study.

### 2.3. Plant Materials

All selected plants were collected from Chiang Mai Province in Thailand during June to December in 2014. The voucher specimens of the collected plants were kept at MANOSÉ Health and Beauty Research Center (http://www.manose.co), Chiang Mai, Thailand. The plant materials were washed, cut into small pieces, dried at 50°C, and then ground to powder.

### 2.4. Preparation of the Plant Extracts

The plant powder was weighed at 20 g. The extracts were prepared from each plant according to the methods indicated in the recipes (containing the selected plants) (methods A to D). The obtained extracts were calculated for yields, kept in glass bottles, and stored at 4 ± 2°C until use. The details of each preparation method indicated in the recipes were as follows.

*Method A: The Recipes Were Orally Administered Together with Honey or Molded as Traditional Pills*. Briefly, the plant powder was mixed with 300 mL of distilled water. The mixtures were stirred for 60 min and centrifuged at 9,000*g* in room temperature (27 ± 2°C) for 5 min. The supernatants were filtered through Whatman™ no. 1 filter paper (GE Healthcare UK Limited, Buckinghamshire, UK). The filtrates were concentrated by a rotary evaporator (BÜCHI Labortechnik AG, Flawil, Switzerland) at 50 ± 2°C and lyophilized by a freeze dryer (Christ Alpha 1-2 LD, Martin Christ Gefriertrocknungsanlagen GmbH, Osterode am Harz, Germany). 

*Method B: The Recipes Were Boiled with Water*. Briefly, the plant powder was mixed with 300 mL of distilled water and boiled for 15 min. After cooling, the mixtures were centrifuged at 9,000*g* in room temperature (27 ± 2°C) for 5 min. The supernatants were filtered through Whatman no. 1 filter paper (GE Healthcare UK Limited, Buckinghamshire, UK). The filtrates were concentrated by a rotary evaporator (BÜCHI Labortechnik AG, Flawil, Switzerland) at 50 ± 2°C and lyophilized by a freeze dryer (Christ Alpha 1-2 LD, Martin Christ Gefriertrocknungsanlagen GmbH, Osterode am Harz, Germany). 

*Method C: The Recipes Were Used as an Infusion*. Briefly, the plant powder was mixed with 300 mL of hot distilled water and stirred for 15 min. After cooling, the mixtures were centrifuged at 9,000*g* in room temperature (27 ± 2°C) for 5 min. The supernatants were filtered through Whatman no. 1 filter paper (GE Healthcare UK Limited, Buckinghamshire, UK). The filtrates were concentrated by a rotary evaporator (BÜCHI Labortechnik AG, Flawil, Switzerland) at 50 ± 2°C and lyophilized by a freeze dryer (Christ Alpha 1-2 LD, Martin Christ Gefriertrocknungsanlagen GmbH, Osterode am Harz, Germany). 

*Method D: The Recipes Were Orally Administered as a Herbal Liquor*. Briefly, the plant powder was mixed with 300 mL of 40% ethanol in distilled water and stirred for 60 min. The mixtures were centrifuged at 9,000*g* in room temperature (27 ± 2°C) for 5 min. The supernatants were filtered through the Whatman no. 1 filter paper (GE Healthcare UK Limited, Buckinghamshire, UK). The filtrates were concentrated by a rotary evaporator (BÜCHI Labortechnik AG, Flawil, Switzerland) at 50 ± 2°C and lyophilized by a freeze dryer (Christ Alpha 1-2 LD, Martin Christ Gefriertrocknungsanlagen GmbH, Osterode am Harz, Germany).

### 2.5. PDEs Activity Inhibition Assay

The PDEs activity inhibition of the selected plant extracts was examined by the modified method [7]. Briefly, 10 *μ*L of the plant extract in distilled water at 1.0 mg/mL was mixed with 20 *μ*L of 0.5 mM 3′,5′-cGMP substrate solution, 5 *μ*L of PDE assay buffer, 10 *μ*L of 5 kU/*μ*L 5′-nucleotidase solution, and 5 *μ*L of 4 U/mL PDE solution in each well of the 96-well half-area microplates (Greiner Bio-One GmbH, Frickenhausen, Germany). The mixtures were incubated at 37 ± 1°C for 60 min. The reaction was stopped by adding 100 *μ*L of the green assay reagent and the mixtures were left to settle at room temperature (27 ± 2°C) for 20 min. The absorbance of the mixtures was measured at 620 nm by a microplate reader (Bio-Rad Laboratories, California, USA). Since the PDEs activity assay was evaluated by measuring the phosphate amounts from the reaction, the amounts of the phosphate existing in the extracts were determined by using the control system of mixing 10 *μ*L of the extract solution with 40 *μ*L of distilled water in each well of the 96-well half-area microplates. The mixtures were incubated at 37 ± 1°C for 60 min and added with 100 *μ*L of the green assay reagent. After keeping at room temperature (27 ± 2°C) for 20 min, the absorbance of the mixtures was measured at 620 nm by a microplate reader. The inhibition of PDE activity was calculated by the following equation:

(1) % inhibition  of  PDEs  activity=ARwater−ARsample−ACsampleARwater×100,

where AR_water_ and AR_sample_ were the absorbance at 620 nm of the mixtures obtained from the reaction of PDEs with distilled water and the plant extracts, respectively; AC_sample_ was the absorbance at 620 nm of the control system to determine the phosphate contents existing in the plant extracts. Each experiment was carried out in triplicate. To determine the concentrations of the extracts giving 50% inhibition of the PDEs activity (IC_50_), the concentration-activity curve was prepared by varying the concentrations of the extracts from 2 × 10^−5^ to 2 × 10^−1 ^mg/mL. Distilled water and sildenafil citrate dissolved in distilled water were used instead of the extracts for the negative and positive controls, respectively. Folds of the PDEs activity inhibition in comparison to sildenafil citrate were calculated as the following equation:

(2) Folds  of  the  PDEs  inhibition  activity=IC50  of  sildenafil  citrateIC50  of  the  samples.

### 2.6. Nitric Oxide Release Stimulation Assay

#### 2.6.1. Cell Culture

The human endothelial cells (EA.hy926) from American Type Culture Collection (ATCC) were cultured in the complete DMEM supplemented with 10% of FBS, 100 U/mL of penicillin, and 100 *μ*g/mL of streptomycin. The cell culture was incubated at 37°C in a humidified 5% CO_2_ incubator (Contherm Scientific Ltd., Petone, New Zealand). The culture medium was changed 2-3 times per week.

#### 2.6.2. Cytotoxicity Assay

The cytotoxicity of the plant extracts on the Ea.hy926 cells was determined by the sulforhodamine B (SRB) assay. Briefly, the EA.hy926 cells (1 × 10^5^ cells/well) in 180 *μ*L of the complete DMEM were added to each well of 96-well microplate and incubated at 37°C for 24 h in a humidified 5% CO_2_ incubator (Contherm Scientific Ltd., Petone, New Zealand). The medium was replaced with the DMEM (without the FBS) containing the plant extracts with the final concentration of 1.0 mg/mL and incubated for 1 h. The medium was decanted out and the new complete DMEM was added and further incubated for 24 h. The cells were fixed with 50 *μ*L/well of the 50% trichloroacetic acid solution and incubated at 4°C for 1 h. The fixed cells were rinsed for five times with distilled water and dried at room temperature (27 ± 2°C). After 24 h, the cells were stained with 100 *μ*L/well of the 0.4% SRB solution and incubated at room temperature (27 ± 2°C) for 30 min. The excess SRB was removed. The cells were washed for 5-6 times with 1% acetic acid solution and dried at room temperature (27 ± 2°C) overnight. The staining SRB in the cells was dissolved by adding 100 *μ*L/well of the 10 mM Tris-base solution with shaking for 30 min. The absorbance was measured at 540 nm by a microplate reader (Bio-Rad Laboratories, California, USA) and the percentage of viable cells was calculated according to the following equation:

(3) Percentages  of  viable  cells=A540  of  the  samplesA540  of  the  control×100,

where *A*_540_ was the absorbance at 540 nm and the control was the untreated cells.

#### 2.6.3. Sample Treatment and Nitric Oxide Measurement

The nitrite concentration was measured using the Griess reaction as described by Green et al. [8]. The EA.hy926 cells (1 × 10^5^ cells/well) were plated in each well of 96-well microplate and incubated at 37°C for 24 h in a humidified 5% CO_2_ incubator (Contherm Scientific Ltd., Petone, New Zealand). The cultured cells were treated with 100 *μ*L of the plant extract, acetylcholine chloride (a positive control), and L-NAME (a negative control) at 1.0 mg/mL in DMEM-no phenol red medium. After incubation at 37°C for 24 h, 100 *μ*L of the cultured cell supernatants was mixed with an equal volume of the Griess reagent and incubated at room temperature (25°C) for 10 min. The absorbance was measured at 540 nm using a microplate reader (Bio-Rad Laboratories, California, USA). The NO production of the EA.hy926 cells was calculated from the obtained absorbance by comparing with the standard concentration curve of sodium nitrite (NaNO_2_) at 1–200 *μ*M in the DMEM-no phenol red medium. Each experiment was carried out in triplicate. To determine the concentrations of the extracts giving 50% stimulation of the NO production activity (SC_50_), the concentration-activity curve was prepared by varying the extract concentrations from 1 × 10^−4^ to 1.0 mg/mL. Folds of the NO release stimulation of the extract in comparing to acetylcholine chloride were calculated as the following equation:

(4) Folds  of  the  NO  release  stimulation  activity=SC50  of  acetylcholine  chlorideSC50  of  the  samples.

### 2.7. Phytochemical Determination

The plant extracts were analyzed for the presence of phytochemicals according to the standard procedures as previously described [9, 10]. Briefly, alkaloids, anthraquinones, carotenoids, flavonoids, glycosides, saponins, tannins, and xanthones were detected by Dragendorff's reagent, ammonium hydroxide (NH_4_OH), sulfuric acid, Shinoda test, thin layer chromatography (TLC), Frothing test, ferric chloride (FeCl_3_), and potassium hydroxide (KOH), respectively.

### 2.8. Acute Oral Toxicity

#### 2.8.1. Animals

Eight-week-old male ICR mice weighing between 25 and 40 g were obtained from the National Laboratory Animal Center, Nakhon Pathom, Thailand. The mice were maintained under the standard condition for laboratory animal housing controlled temperature at 24 ± 1°C and humidity at 50 ± 10% with the cycle of 12 h light and 12 h dark. The mice were fed with commercially available animal foods and allowed water ad libitum. All experiments were conducted in accordance with the directive 2010/63/EU and the guidelines for the care and use of laboratory animals of Chiang Mai University, Chiang Mai, Thailand. The date and number from the ethical committee of the Laboratory Animal Center, Chiang Mai University, for the in vivo experiment were 28/01/2016 and 002/2559[01/2559-01-28], respectively.

#### 2.8.2. Acute Oral Toxicity Test

The acute oral toxicity of the selected plant extracts was performed according to the Organization Economic Co-operation and Development (OECD) guideline for testing of chemicals: Acute Oral Toxicity: Up-and-Down Procedure (OECD guideline number 425) [11]. The male mice were weighed and randomly divided into two groups (5 mice per group). The animals were orally given using an oral feeding tube the vehicle (distilled water) as a control group in Group I and in Group II a single dose of the selected plant extract at 5,000 mg/kg body weight. Mortality and toxicity apparent signs or behavioral alterations of mice were observed daily for 14 days. The body weight changes of the mice were checked at day 14. At the end of the experiment, each mouse was euthanized and the organs (heart, lung, liver, kidney, spleen, stomach, intestine, and testis) were collected for histopathology. The organs were isolated and preserved in 10% formalin in phosphate buffered saline (PBS) at pH 7.4. Each organ was weighed and the abnormal-weight organs were embedded in paraffin, cut into 6 and 12 *μ*m thickness, stained with hematoxylin and eosin, and examined under a light microscope (Olympus, Tokyo, Japan).

### 2.9. Evaluation of Sexual Behavior Stimulatory Effects

#### 2.9.1. Animals

Healthy eight-week-old ICR male and female mice weighing 35–40 g and 30–35 g, respectively, were obtained from the National Laboratory Animal Center, Nakhon Pathom, Thailand. The mice were maintained under the standard condition for laboratory animal housing controlled temperature at 24 ± 1°C and humidity at 50 ± 10% with the cycle of 12 h light and 12 h dark. The mice were fed with commercially available animal foods and allowed water ad libitum.

#### 2.9.2. Induction of Estrous Period to the Female Mice

The female mice were induced to be in the estrous period by the procedure modified from the method of Aslam and Sial [12]. Briefly, the female mice were orally given 17*α*-ethinylestradiol at 2 mg/kg body weight and subcutaneously injected with progesterone at 20 mg/kg body weight, 48 h and 6 h, respectively, before exposure to the male mice. The estrous period of the female mice was confirmed by vaginal smear examinations under microscopic observation [13].

#### 2.9.3. Induction of Sexual Dysfunction or ED (Erectile Dysfunction) of the Male Mice

The male mice were induced to be in the state of sexual dysfunction by daily oral administration for 21 days of paroxetine suspension at 20 mg/kg body weight using a metal feeding tube [14]. The paroxetine suspension was prepared daily in Tween-80 suspended in normal saline solution. After 21 days, an estrous female mouse was introduced into each paroxetine-treated male mouse in a separate cage, and the sexual behaviors including numbers of courtship (NC), mount frequency (MF), intromission frequency (IF), and ejaculatory frequency (EF) were observed via a video record for 12 h between 6.00 p.m. and 6.00 a.m. The male mice which showed the minimum of 25% reduction in NC, MF, IF, and EF when compared with the normal male mice were considered as sexually dysfunctional and were used for the further study. The observing staffs were free from treatment information and have evaluated the male sexual behaviors according to the criteria of the previous studies [15, 16] as follows:

- Courtship: it is the amount of frequency it takes for a male to mount the female for the first time.
- Mounting the female: the male normally mounts from the rear, sometimes posing his forelegs over the female's back, and rapidly makes.
- Intromission vaginal penetration: this behavior starts with a mount, but suddenly the male makes a deep thrust forward and stops pelvic thrusting.
- Ejaculation: this behavior starts with an intromission, but, after vaginal penetration (the deep forward thrust), the male remains on the female for 1–3 s.

#### 2.9.4. Animal Grouping and Extract Administration

The paroxetine-induced sexual dysfunction male mice were randomly divided into three groups with 3 animals in each group. 300 *μ*l of distilled water, 0.02 g/kg body weight of sildenafil (a standard PDE-5 inhibitor drug), and 1 g/kg body weight of the plant extract were orally administered to each animal group using a metal oral feeding tube, once daily for 14 days. After 30 mins of administration, an estrous female mouse was introduced into each sample-treated male mouse in a separate cage and the sexual behaviors of the male mice were monitored at days 1, 7, and 14. The sexual behaviors including NC, MF, IF, and EF were observed via a video record for 12 h between 6.00 p.m. and 6.00 a.m.

### 2.10. Statistical Analysis

All assays were performed in triplicate of independent experiments. The data were presented as means ± standard deviation (SD). Statistical evaluation was determined by the one-way analysis of variance (one-way ANOVA) with post hoc test of multiple comparisons. Statistical significance was considered at the *p* values of less than 0.01 and 0.05.

## 3. Results and Discussion

### 3.1. Selection of Plants from the Thai Medicinal Plant Recipe Database “MANOSROI III”

Seven instead of six medicinal plants in the top three ranks searching by the two Thai keywords (“Sueam Sa Mat Tha Phap Thang Phet” and “Bam Rung Kam Lang Thang Phet”) were obtained and used to prepare the extracts according to the details shown in Table 1, since there were two medicinal plants that gave the same frequency in the selected aphrodisiac recipes.

### 3.2. Preparation of the Extracts

The extracts of the seven selected medicinal plants were prepared according to the traditional methods (methods A–D) from the recipes containing these plants. Fifteen extracts were obtained because some selected plants were from the recipes which indicated more than one traditional preparation method. The percentage yields and physical appearance of the extracts were demonstrated in Table 1. Methods A–C used water as a solvent, while method D used ethanol as a solvent. Six out of 15 extracts were prepared by method A which was the traditional indication of most Thai medicinal plant recipes. The six extracts gave the percentage yields of 2.55–22.90%, while the extract of* Plumbago indica* L. demonstrated the highest percentage yield of 22.90%. However, the extract of* P. indica* prepared by method C gave the highest percentage yield of 26.60% compared to other extracts. This may be due to the use of hot water to prepare the extract in which the phytochemical compounds could be dissolved better than in cold water.

### 3.3. PDEs Activity Inhibition of the Selected Plant Extracts

The dose of 15 extracts at 200 *μ*g/mL which was calculated from the traditional dose indicated in the recipes was tested for PDEs inhibition compared with sildenafil, a positive control PDE-5 inhibitor drug. The dose of sildenafil at 0.45 *μ*g/mL was determined from the maximum plasma concentration of a single oral dose of tablet containing 100 mg sildenafil in healthy male volunteers [17]. The IC_50_ values and PDEs inhibition percentages of the extracts and sildenafil were shown in Table 2. All extracts [EDP1-001(1), EDP1-001(2), EDP1-002(2), EDP1-003, EDP2-001(1), EDP2-002(2), and EDP2-003(2)] exhibited the PDEs inhibition percentages in the range of 2.72–65.28% of 0.18–4.36-fold sildenafil. Sildenafil provided quite low inhibitory activity to PDEs because the dose of sildenafil (0.45 *μ*g/mL) used in the test was lower than the extracts (200 *μ*g/mL). The IC_50_ value of sildenafil was higher than EDP1-001(1). Also, sildenafil is a selective inhibitor of phosphodiesterase (PDE) type 5, but the nonselective phosphodiesterases (PDEs) assay was performed in this study. So, the different potency of PDEs inhibition activity between EDP1-001(1) and the standard sildenafil may be due to the different inhibition mechanism on PDEs. The mechanism of sildenafil is the potential endogenous increases in cGMP by inhibiting its breakdown at the catalytic site [18]. Orallo et al. [19] showed that (±) naringenin as a natural flavonoid characterized as an agent with clear vasorelaxant effects on rat aortic smooth muscle is probably mediated by an increase in systolic cAMP and cGMP concentrations. However, some plant extracts showed PDEs stimulation effect (with the minus PDEs stimulation value). This may be due to affinity to noncatalytic CGMP blinding sites on the PDEs [20]. The highest PDEs inhibition percentage was observed in EDP1-001(1) extract at 65.28 ± 2.09% of 4.36-fold sildenafil. This extract gave the IC_50_ value of PDEs inhibition activity at 0.0026 ± 0.0025 mg/mL of 2.23-fold sildenafil (the IC_50_ value of sildenafil was 0.0058 ± 0.0032 mg/mL). This extract was prepared by method A using aqueous cold extraction of* Boesenbergia rotunda* (L.) Mansf. rhizome (Table 1). As previously reported, the ethanolic extract of this plant at 0.1 mg/mL showed complete inhibitory effect (100% inhibition) against PDEs [21]. This ethanolic extract at 50 *μ*g/mL also exhibited the selective inhibition against PDE-5 activity of 40.86 ± 3.94% [22]. In addition, this present study has also demonstrated high PDEs inhibition activity of EDP1-002(2), EDP2-003(2), EDP1-003, and EDP0-001(2) which were extracts from* Piper nigrum* Linn.,* Piper interruptum* Opiz,* Diospyros rhodocalyx* Kurz., and* Plumbago indica* Linn., respectively. Although sildenafil is a PDE5 inhibitor, the common side effects such as headache, flushing, dizziness, nasal congestion, and dyspepsia, which are a reflection of the vasodilatory effects on the capillary smooth muscle in other parts of the body, have been reported. So far, the inhibitory selectivity on PDE5 of the extract from* B. rotunda* has not been investigated. However, this extract was obtained from* B. rotunda* which is a Thai medicinal plant that has long been traditionally consumed. In addition, Saraithong et al. [23] have demonstrated that the ethanolic extract of* B. rotunda* was safe for consumption as in vivo studies of* B. rotunda* fed to rats showed no significant changes in body weight and all hematological and histopathological parameters used to evaluate the toxicity effect did not show any adverse changes. Thus, the extract from* B. rotunda* is safe for consumption. This result has indicated the enhancing sexual performance potential of the Thai medicinal plants searched from the Thai medicinal plant recipe database “MANOSROI III,” which have long been traditionally used in humans. In fact, this long traditional use in humans is similar to the clinical trial step in the research and development of the modern drugs [6].

### 3.4. NO Release Stimulation Activity of the Selected Plant Extracts

**Figure 1:**
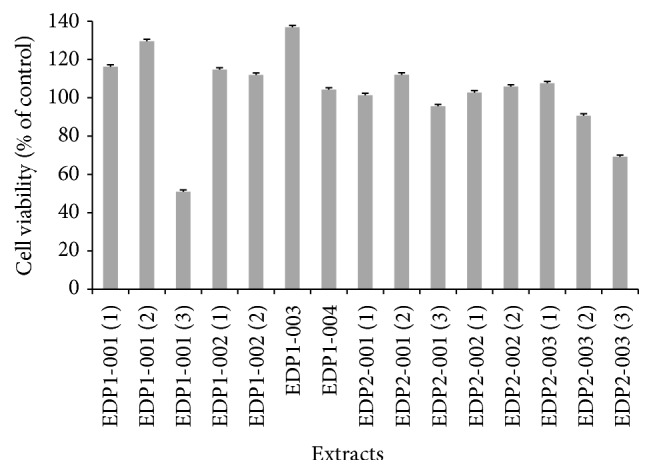
Cell viability of EA.hy926 treated with the 15 selected plant extracts at 1.0 mg/mL [% cell viability = (*A*_540_ of the sample/*A*_540_ of the control) × 100, where *A*_540_ was the absorbance at 540 nm and the control was the untreated cells].

The NO released from endothelial cells is a major endogenous vasodilator system for counterbalancing the vasoconstriction produced by the sympathetic nervous system and the rennin-angiotensin system [24]. In Figure 1, the cell viability percentages of EA.hy926 treated with all selected plant extracts, except EDP1-001(3) and EDP2-003(3) at 1.0 mg/mL, were close or more than 100% of the control. Only EDP1-001(3) and EDP2-003(3) at 1.0 mg/mL exhibited cell viability of less than 70% of the control. Thus, the extract at 1.0 mg/mL was used for the NO release stimulation assay. In Table 2, ten out of 15 selected plant extracts including EDP1-001(1), EDP1-001(2), EDP1-001(3), EDP1-002(1), EDP1-003, EDP1-004, EDP2-001(1), EDP2-001(2), EDP2-001(3), and EDP2-002(2) showed the NO release stimulation activity in the range of 33.53–666.85%. EDP2-001(1) exhibited the highest NO release stimulation activity of 666.85 ± 24.66% with 1.50-fold the acetylcholine (a positive control, which gave the activity of 443.63 ± 91.94%). However, EDP1-002(2), EDP2-002(1), EDP2-003(1), EDP2-003(2), and EDP2-003(3) extracts displayed the NO release inhibition the same as L-NAME (a NO release inhibitor, a negative control), indicating the inhibition of NO production. The EDP1-001(1) and EDP1-003 extracts exhibited the SC_50_ values of NO release stimulation at 3.96 ± 5.57 and 0.49 ± 0.33 mg/mL of 0.002- and 0.016-fold acetylcholine (the SC_50_ value of acetylcholine was 0.008 ± 0.003 mg/mL), respectively. EDP1-001(1) and EDP1-003 extracts were prepared by method A from* Boesenbergia rotunda* (L.) Mansf. and* Diospyros rhodocalyx* Kurz., respectively. High quantity of flavonoids at 12,975.52 ± 71.78 *μ*g/g of the dry weight of the* B. rotunda* extract has been reported [25]. Both flavonoids and phenolic compounds have been found in the* D. rhodocalyx* extract [26]. The flavonoid and phenolic compounds have been previously reported to exhibit NO production stimulation in the EA.hy926 cells [27, 28]. Wang et al. have presented that the flavonoids in Shxiao San extract augmented the NO output in the EA.hy926 cells [28]. The treatment of EA.hy926 cells with an alcohol-free red wine polyphenol extract showed an increase of the NO release [29]. Thus, the NO release stimulation of the EDP1-001(1) and EDP1-003 extracts might be from the flavonoid and phenolic contents.

### 3.5. Phytochemical Determination of the Selected Plant Extracts

**Figure 2:**
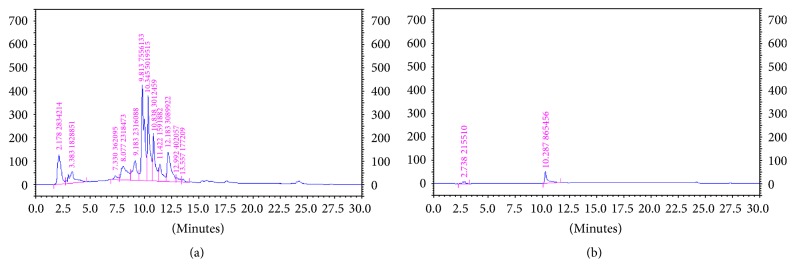
HPLC fingerprint of the EDP1-001(1) extract at 100 mg/ml (a) which exhibited the peak of flavone at the retention time of 10.345 min with the content of 58 *µ*g/mg extract and HPLC fingerprint of the standard flavone at 1 mg/ml (b).

The phytochemical contents in the selected plant extracts were presented in Table 3. High contents of flavonoids and saponins were found in EDP1-001(1), whereas alkaloids and xanthones (phenolic compounds) were observed in EDP1-003. Bioactive compounds from rhizome extracts of* B. rotunda* have been classified mainly into two major groups including flavanones (alpinetin, pinostrobin, and pinocembrin) and chalcones (boesenbergin, cardamonin, panduratin A, and 4-hydroxypanduratin A) [30]. These compounds have estrogenic or androgenic activities and show phosphodiesterase inhibition activity [31]. Some plants have demonstrated PDEI activity related to their saponin contents. Mimaki et al. [32] reported that* Lilium regale* and* Lilium henryi* have inhibitory effects on cAMP PDE. The main active substances presented in these plants are steroidal saponins. Kuroda et al. [33] showed that ethanol extract of* Allium chinense* has inhibitory activity on cAMP PDE, probably because of its saponin contents. These major bioactive compounds can be used as markers for phytochemical profile standardization. In this study, the HPLC fingerprint of the EDP1-001(1) extract using flavone as a marker was performed for quality control, with the flavone content of 58 *μ*g/mg extract (Figure 2). Both flavonoids and phenolic compounds have been reported to be related to NO production stimulation [27–29], with the possible mechanism of increasing mRNA and protein expression of endothelial NO synthase (eNOS) in rats [34]. NO might be stimulated and released directly in the endothelial cells by activating the eNOS. Upadhyaya et al. have reported that flavonoids in Shixiao San increased the expression of eNOS [35]. In addition, an increase in the eNOS mRNA expression of the endothelial cells could be observed after being incubated with red wine polyphenol extract [29]. Thus, flavonoid contents in EDP1-001(1) appeared to be responsible for the NO release stimulation activity.

### 3.6. Acute Oral Toxicity

The EDP1-001(1) extract was further investigated for acute oral toxicity in male mice because it gave the highest activity of PDEs inhibition and high NO release stimulation activity with no cytotoxicity in the EA.hy926 cells (Table 4). Mortality, abnormal animal behaviors, toxic signs, and body weight changes compared to the control group of the male mice fed with a single dose of 5,000 mg/kg body weight were not observed. Hence, the median lethal dose (LD_50_) of the EDP1-001(1) extract was more than 5,000 mg/kg body weight. In addition, no macroscopic change of the internal organs of all treated mice was indicated. Most internal organ weights of the extract-treated mice were not different from the control group, except the liver and intestine weights which increased significantly (*p* < 0.05) when compared with the control group. However, the histopathological examination of these two organs was not different from the control group. Therefore, the* B. rotunda* extract [EDP1-001(1)] was safe and appeared to have potential for the further development as a product.

### 3.7. Sexual Behavior Stimulatory Effects

Sildenafil at 0.02 g/kg body weight was calculated from the normal dose of sildenafil in human (100 mg/60 kg body weight) and adjusted by the dose conversion factor to mice [36]. The selected plant extract, EDP1-001(1), at 1 g/kg body weight was obtained from the traditional indication in the recipe and also adjusted by the conversion factor from human to mice. The sexual behaviors including number of courtships (NC), mount frequency (MF), intromission frequency (IF), and ejaculatory frequency (EF) of the paroxetine-induced sexual dysfunction male mice treated with the EDP1-001(1) extract compared to the control groups were shown in Tables 5 and 6. The paroxetine-induced sexual dysfunction male mice that were orally given daily the EDP1-001(1) extract for 7 and 14 days exhibited sexual behavior improvement when compared with the mice treated with distilled water (the control group). At day 7 of the treatment, the NC and MF of the EDP1-001(1)-treated mice were at 26.33 ± 3.48 and 17.00 ± 12.34 which were 1.44- and 17.00-fold the distilled water-treated mice (the control group), respectively. At day 14 of the treatment, the NC, MF, IF, and EF of the EDP1-001(1)-treated mice were at 87.67 ± 6.17, 121.00 ± 23.50, 36.00 ± 3.21, and 13.67 ± 2.96 which were 2.63-, 1.27-, 0.53-, and 0.62-fold the sildenafil-treated mice (the positive control group), respectively. The EDP1-001(1) appeared to be a potential extract for the treatment of sexual dysfunction. However, the extract needed to be orally administered continuously for 7–14 days to obtain an improvement of sexual dysfunction symptoms, whereas sildenafil exhibited the improvement particularly at the initial treatment of day 1. The higher sexual behavior stimulatory effects of the EDP1-001(1) extract at 1 g/kg body weight than sildenafil at 0.02 g/kg body weight might be due to the regulatory mechanism of male reproductive system that are not related only to the inhibition of PDEs activity, but also to the influence on androgens such as testosterone and the release of NO. This in vivo sexual dysfunction improvement of the EDP1-001(1) extract has confirmed the in vitro results of both the PDEs inhibition and the NO release stimulation with the IC_50_ value of 0.0026 ± 0.0025 mg/mL (2.23-fold sildenafil) and the SC_50_ value of 3.96 ± 5.57 mg/mL (0.002-fold acetylcholine), respectively.

## 4. Conclusions

Several Thai medicinal plants appeared in the Thai medicinal plant recipe database “MANOSROI III” have long been traditionally used to treat various diseases and symptoms including ED. Seven aphrodisiac medicinal plants selected from the top three ranks searched from the “MANOSRO III” database by the 2 Thai keywords (“Sueam Sa Mat Tha Phap Thang Phet” and “Bam Rung Kam Lang Thang Phet”) were prepared as 15 extracts. Only 7 extracts at 200 *μ*g/mL exhibited the PDEs inhibition in the range of 2.72–65.28% which were 0.18–4.36-fold sildenafil at 0.45 *μ*g/mL, while 10 out of 15 extracts at 1.0 mg/mL showed the NO release stimulation in the range of 33.53–666.85% in the EA.hy926 cells which were 0.08–1.50-fold acetylcholine at 1 mg/mL. The EDP1-001(1) extract prepared from* B. rotunda* by method A gave the highest PDEs inhibition activity with the IC_50_ value of 0.0026 ± 0.0025 mg/mL and the high NO release stimulation with the SC_50_ value of 3.96 ± 5.57 mg/mL. The EDP1-001(1) extract contained the high contents of flavonoids and saponins in which flavonoids have been reported to be related to NO production stimulation. The EDP1-001(1) was nontoxic to EA.hy926 cells at 1.0 mg/mL. The EDP1-001(1) extract showed no acute toxicity with no mortality, no abnormal animal behaviors, no toxic signs, and no body weight changes after oral administration in male ICR mice at a single dose of 5,000 mg/kg body weight. This extract also exhibited the sexual behavior improvement in the paroxetine-induced sexual dysfunction male mice after oral daily administration for 14 days when compared with the control group. At day 14 of the treatment, the NC, MF, IF, and EF of the EDP1-001(1)-treated mice were at 87.67 ± 6.17, 121.00 ± 23.50, 36.00 ± 3.21, and 13.67 ± 2.96 which were 2.63-, 1.27-, 0.53-, and 0.62-fold the sildenafil-treated mice (positive control group), respectively. The results from this study have indicated not only the potential of the selected Thai plant extract for the further development as food supplements for ED but also the benefit of the Thai medicinal plant recipe database “MANOSROI III” as a convenient source for an efficient searching and ranking of the target plants for the treatment of various symptoms including ED.

## Figures and Tables

**Table 1:** Seven medicinal plants selected from the Thai medicinal plant recipe database “MANOSROI III” and their extracts.

Number	Medicinal plants				Preparation of the extracts			
	Code	Botanical name	Family	Part used	Recipe preparation indication	Extract code	% yield (w/w)	Physical appearance of the extracts
1	EDP1-001	*Boesenbergia rotunda* (L.) Mansf.	Zingiberaceae	Rhizome	A	EDP1-001(1)	10.80	Brown, slightly viscous semisolid
					B	EDP1-001(2)	8.15	Light brown, powder
					D	EDP1-001(3)	6.85	Brown, slightly viscous semisolid

2	EDP1-002	*Piper nigrum* Linn.	Piperaceae	White seed	A	EDP1-002(1)	2.55	Dark brown, viscous semisolid
					B	EDP1-002(2)	1.45	Light brown, powder

3	EDP1-003	*Diospyros rhodocalyx* Kurz.	Ebenaceae	Whole	A	EDP1-003	6.60	Dark brown, viscous semisolid

4	EDP1-004	*Piper nigrum* Linn.	Piperaceae	Black seed	A	EDP1-004	4.30	Dark brown, viscous semisolid

5	EDP2-001	*Plumbago indica* Linn.	Plumbaginaceae	Whole	A	EDP2-001(1)	22.90	Dark brown, slightly viscous semisolid
					C	EDP2-001(2)	26.60	Dark brown, viscous semisolid
					D	EDP2-001(3)	24.20	Red-brown, slightly viscous semisolid

6	EDP2-002	*Piper nigrum* Linn.	Piperaceae	Black seed	C	EDP2-002(1)	5.60	Dark brown, slightly viscous semi-solid
					D	EDP2-002(2)	5.20	Dark brown, slightly viscous semisolid

7	EDP2-003	*Piper interruptum* Opiz	Piperaceae	Whole	A	EDP2-003(1)	16.30	Dark brown, slightly viscous semisolid
					B	EDP2-003(2)	6.45	Dark brown, slightly viscous semisolid
					D	EDP2-003(3)	15.00	Red-brown, viscous semisolid

*Method* A: the recipes were orally administered together with honey or molded as traditional pills. *Method* B: the recipes were boiled with water. *Method* C: the recipes were used as an infusion. *Method* D: the recipes were orally administered as a herbal liquor.

**Table 2:** Phosphodiesterases (PDEs) inhibition and nitric oxide (NO) release stimulation activities of the fifteen selected plant extracts.

Number	Samples	Inhibition of PDEs activity				Stimulation of NO release			
		%		IC_50_ (mg/mL)		%		SC_50_ (mg/mL)	
		Mean ± S.D.	Folds of sildenafil	Mean ± SD	Folds of sildenafil	Mean ± SD	Folds of acetylcholine	Mean ± SD	Folds of acetylcholine
1	EDP1-001(1)	65.28 ± 2.09	4.36	0.0026±0.0025	2.23	100.17 ± 11.24	0.23	3.96 ± 5.57	0.002
2	EDP1-001(2)	10.35 ± 5.22	0.69	—	—	33.53 ± 12.69	0.08	—	—
3	EDP1-001(3)	−667.44 ± 64.76	−44.61	—	—	129.99 ± 49.70	0.29	—	—
4	EDP1-002(1)	−22.22 ± 10.03	−1.49	—	—	122.37 ± 27.91	0.28	—	—
5	EDP1-002(2)	63.74 ± 2.28	4.26	—	—	−104.10 ± 5.80	−0.23	—	—
6	EDP1-003	8.77 ± 5.14	0.59	—	—	162.45 ± 19.57	0.37	0.49 ± 0.33	0.016
7	EDP1-004	−42.75 ± 8.01	−2.86	—	—	275.00 ± 39.08	0.62	—	—
8	EDP2-001(1)	2.72 ± 4.82	0.18	—	—	666.85 ± 24.66	1.50	—	—
9	EDP2-001(2)	7.24 ± 6.71	0.48	—	—	312.13 ± 4.36	0.70	—	—
10	EDP2-001(3)	−16.77 ± 10.69	−1.12	—	—	179.03 ± 11.01	0.40	—	—
11	EDP2-002(1)	−47.40 ± 6.74	−3.17	—	—	−133.32 ± 7.90	−0.30	—	—
12	EDP2-002(2)	−34.06 ± 4.02	−2.28	—	—	277.27 ± 82.65	0.63	—	—
13	EDP2-003(1)	−2.20 ± 3.72	−0.15	—	—	−60.79 ± 9.07	−0.14	—	—
14	EDP2-003(2)	44.02 ± 2.49	2.94	—	—	−110.76 ± 34.08	−0.25	—	—
15	EDP2-003(3)	−3.81 ± 2.08	−0.25	—	—	−115.38 ± 23.21	−0.26	—	—

16	Sildenafil	14.96 ± 8.39	1	0.0058 ± 0.0032	1	—	—	—	—

17	Acetylcholine	—	—	—	—	443.63 ± 91.94	1	0.008 ± 0.003	1

18	L-NAME	—	—	—	—	−85.12 ± 10.47	−0.19	−38.10 ± 16.96	−0.0002

*Inhibition of PDEs activity*: inhibition percentages were determined at the concentrations of the extracts and sildenafil at 200 and 0.45 *µ*g/mL, respectively. IC_50_ was the concentrations of samples giving 50% inhibition of the PDEs activity. The minus value indicated the stimulation of PDEs. * Stimulation of NO release*: stimulation percentages of the extracts, acetylcholine, and L-NAME were tested at the concentration of 1 mg/mL. SC_50_ was the concentrations of samples giving 50% stimulation of the NO production activity. L-NAME was N_*ω*_-nitro-L-arginine methyl ester hydrochloride (NO release inhibitor). The minus value indicated the inhibition of NO production.

**Table 3:** Phytochemicals of the selected plant extracts.

Phytochemical contents	Extracts	
	EDP1-001(1)	EDP1-003
Alkaloids	—	+
Anthraquinones	—	—
Carotenoids	—	—
Flavonoids	+++	—
Glycosides	—	—
Saponins	+++	—
Tannins	—	—
Xanthones	—	+

+++: abundant presence, +: slight presence; —: absence.

**Table 4:** Body and organ weights of the male mice (*n* = 5) after being orally given the EDP1-001(1) extract at the single dose of 5,000 mg/kg body weight.

Samples	Body weight (g)		Organ weights (g) at day 14							
	Before	Day 14	Heart	Lung	Liver	Spleen	Kidneys	Stomach	Intestine	Testis
EDP1-001(1)	38.33 ± 2.58	47.10 ± 2.37	0.29 ± 0.03	0.36 ± 0.02	2.80 ± 0.18^*∗*^	0.16 ± 0.05	0.85 ± 0.04	0.34 ± 0.04	4.01 ± 0.66^*∗*^	0.32 ± 0.03
Water (control)	41.60 ± 2.58	41.67 ± 2.58	0.28 ± 0.03	0.42 ± 0.04	2.39 ± 0.20	0.15 ± 0.02	0.91 ± 0.07	0.36 ± 0.05	3.10 ± 0.29	0.34 ± 0.04

^*∗*^Significant difference (*p* < 0.05) in comparison to the control group.

**Table 5:** Sexual behavior stimulatory effects on numbers of courtship and mount frequency in paroxetine-induced sexual dysfunction male mice (*n* = 3) after daily oral administration of the EDP1-001(1) extract for 1, 7, and 14 days.

Day	Numbers of courtship					Mount frequency				
	Distilled water	Sildenafil	EDP1-001(1) extract			Distilled water	Sildenafil	EDP1-001(1) extract		
			Number	Fold_1_	Fold_2_			Frequency	Fold_1_	Fold_2_
1	16.00 ± 4.62	56.00 ± 5.69	12.33 ± 7.84	0.77	0.22	28.33 ± 14.43	79.67 ± 46.19	16.33 ± 10.48	0.58	0.01
7	18.33 ± 3.76	27.00 ± 17.09	26.33 ± 3.48	1.44	0.98	1.00 ± 1.00	101.00 ± 2.08	17.00 ± 12.34	17.00	0.17
14	15.67 ± 2.85	33.33 ± 2.73	87.67 ± 6.17	5.60	2.63	1.33 ± 0.88	95.33 ± 4.41	121.00 ± 23.50	90.75	1.27

Fold_1_ was calculated by comparing with the control group (orally administered with distilled water). Fold_2_ was calculated by comparing with the positive control group (orally administered with sildenafil).

**Table 6:** Sexual behavior stimulatory effect on intromission frequency and ejaculatory frequency in paroxetine-induced sexual dysfunction male mice (*n* = 3) after daily oral administration of the EDP1-001(1) extract for 1, 7, and 14 days.

Day	Intromission frequency					Ejaculatory frequency				
	Distilled water	Sildenafil	EDP1-001(1) extract			Distilled water	Sildenafil	EDP1-001(1) extract		
			Frequency	Fold_1_	Fold_2_			Frequency	Fold_1_	Fold_2_
1	18.00 ± 11.36	65.67 ± 43.53	4.67 ± 1.00	0.26	0.07	1.33 ± 1.33	7.33 ± 4.67	0	0	0
7	0	64.67 ± 24.13	1.00 ± 0.58	—	0.02	0	16.00 ± 8.50	0	—	0
14	0.33 ± 0.33	68.33 ± 16.05	36.00 ± 3.21	109.09	0.53	0	22.00 ± 2.52	13.67 ± 2.96	—	0.62

Fold_1_ was calculated by comparing with the control group (orally administered with distilled water); Fold_2_ was calculated by comparing with the positive control group (orally administered with sildenafil).
